# Inter-individual body mass variations relate to fractionated functional brain hierarchies

**DOI:** 10.1038/s42003-021-02268-x

**Published:** 2021-06-14

**Authors:** Bo-yong Park, Hyunjin Park, Filip Morys, Mansu Kim, Kyoungseob Byeon, Hyebin Lee, Se-Hong Kim, Sofie L. Valk, Alain Dagher, Boris C. Bernhardt

**Affiliations:** 1 McConnell Brain Imaging Centre, Montreal Neurological Institute and Hospital, McGill University, Montreal, QC Canada; 2 Department of Data Science, Inha University, Incheon, Republic of Korea; 3 School of Electronic and Electrical Engineering, Sungkyunkwan University, Suwon, Republic of Korea; 4 Center for Neuroscience Imaging Research, Institute for Basic Science, Suwon, Republic of Korea; 5 Department of Biostatistics, Epidemiology, and Informatics, University of Pennsylvania, Philadelphia, PA USA; 6 Department of Electrical and Computer Engineering, Sungkyunkwan University, Suwon, Republic of Korea; 7 Department of Family Medicine, St. Vincent’s Hospital, Catholic University College of Medicine, Suwon, Republic of Korea; 8 Otto Hahn Research Group for Cognitive Neurogenetics, Max Planck Institute for Cognitive and Brain Sciences, Leipzig, Germany; 9 INM-7, FZ Jülich, Jülich, Germany

**Keywords:** Network models, Image processing

## Abstract

Variations in body mass index (BMI) have been suggested to relate to atypical brain organization, yet connectome-level substrates of BMI and their neurobiological underpinnings remain unclear. Studying 325 healthy young adults, we examined associations between functional connectivity and inter-individual BMI variations. We utilized non-linear connectome manifold learning techniques to represent macroscale functional organization along continuous hierarchical axes that dissociate low level and higher order brain systems. We observed an increased differentiation between unimodal and heteromodal association networks in individuals with higher BMI, indicative of a disrupted modular architecture and hierarchy of the brain. Transcriptomic decoding and gene enrichment analyses identified genes previously implicated in genome-wide associations to BMI and specific cortical, striatal, and cerebellar cell types. These findings illustrate functional connectome substrates of BMI variations in healthy young adults and point to potential molecular associations.

## Abstract (web summary)

Bo-yong Park et al. use non-linear connectome manifold learning to examine the association between brain connectivity and inter-individual body mass index (BMI) in 325 young adults. They supplement these analyses with existing transcriptomic data, altogether suggesting several neural and molecular associations that may underlie BMI variations in healthy young adults.

## Introduction

A high body mass index (BMI) has been recognized as an important contributor to adverse health and psychological outcomes^1–3^. High BMI is an indicator of obesity, a condition with increasing prevalence worldwide^3^ and a contributing factor to the development of type 2 diabetes, cardiovascular disease, stroke, cancer, and metabolic syndrome^4–7^. In addition, multiple neurobiological processes related to obesity have been recognized, including mechanisms regulating eating behaviors, together with genetic and transcriptomic underpinnings^7–16^.

Neuroimaging techniques, particularly magnetic resonance imaging (MRI), can identify cerebral substrates associated with BMI by tapping into whole-brain structure, function, and connectivity. Prior structural MRI research has shown that measures of cortical and subcortical morphology robustly correlate with inter-individual variations of BMI in healthy^11,17–19^ and diseased populations^20,21^. Multiple task-based functional MRI studies have also shown associations between BMI and brain activations in impulse control and reward processing paradigms^22–29^. During resting conditions, studies reported associations between BMI and connectivity of specific regions^30–33^ and larger networks involved in cognitive control and reward systems^34–36^. A recent study suggested regional functional connectivity patterns related to inter-individual variations in obesity phenotypes using machine learning^37,38^. However, it is less well established how these patterns are associated with whole-brain functional networks. The current work aims to address this gap by applying connectome manifold learning techniques to identify functional substrates of BMI in a large population of healthy adults. The key to manifold learning is the ability to compress high-dimensional connectomes into a series of lower-dimensional eigenvectors (i.e., gradients) that visualize spatial trends in inter-regional connectivity variations^39^, simplifying connectivity analysis and visualization. Eigenvectors estimated from resting-state functional MRI (rs-fMRI), myelin-sensitive imaging, and diffusion MRI can serve as axes of the brain’s intrinsic coordinate system^39–45^. These eigenvectors have been shown to follow established models of neural hierarchy and laminar differentiation^46^. Complementing modular descriptions of brain networks in terms of network integration and segregation, these manifold learning techniques thus offer a data-driven perspective on the gradual and hierarchical organization of functional and structural brain systems in health and disease^39,41,47–51^. In the context of BMI, these techniques have not been applied but promise to assess whether patterns of functional network integration and segregation reflect inter-individual body mass variations.

As the above manifold learning can generate cortical maps capturing large-scale principles of brain connectivity and hierarchical differentiation, these features can be readily integrated with other spatial features of brain organization. Spatial associations between connectome gradients and measures of brain morphology and microstructure can query shared and unique effects. Furthermore, neurobiological data that is not per se neuroimaging derived is increasingly represented in MRI reference space. One such repository, comprising *post-mortem* gene expression maps, has been disseminated by the Allen Institute for Brain Science (AIBS)^52–56^. This resource can inform spatial association analyses between imaging-derived findings and gene expression patterns. Coupled with gene set enrichment analyses^57–61^, these approaches can discover molecular, developmental, and disease-related processes, and provide additional context for MRI findings. Recent studies utilized transcriptomic decoding to explore the underpinnings of brain imaging findings in both healthy and diseased cohorts^45,50,62–66^.

Here, we studied associations between macroscale functional connectome organization and inter-individual variations in BMI. Our functional network analysis was based on the identification of connectome manifolds, which offer a continuous and low dimensional analytical space to interrogate macroscale brain organization and network hierarchy^39,40,67^. Studying the multimodal human connectome project (HCP) dataset^68^, we also examined whether associations between functional manifolds and BMI existed above and beyond structural effects as measured by MRI-based measures of cortical thickness, sulco-gyral folding, and intracortical myelin. To explore neurobiological underpinnings of BMI-related whole-brain connectome changes, we performed spatial association analyses to *post-mortem* gene expression data and carried out gene enrichment analyses.

## Results

We studied 325 unrelated young and healthy adults (mean ± SD age = 28.56 ± 3.74 years; 55% female; mean ± SD BMI = 26.30 ± 5.16 kg/m^2^, range 16.65–47.76 kg/m^2^) from the S900 release of the HCP^68^. Details on participant selection, image processing, and analysis are outlined in the “Methods”. Reproducibility was studied in an additional 74 unrelated healthy adults from the HCP S1200 release (mean ± SD age = 28.08 ± 3.90 years; 34% female; mean ± SD BMI = 26.17 ± 4.39 kg/m^2^, range 18.89–39.47 kg/m^2^), as well as an independent dataset of healthy adults acquired from the St. Vincent’s Hospital (SVH; *n* = 36; mean ± SD age = 38.78 ± 10.52 years; 47% female; mean ± SD BMI = 29.38 ± 6.29 kg/m^2^, range 23.15–57.13 kg/m^2^).

### Macroscale functional manifolds are associated with inter-individual variations in BMI

**Fig. 1:**
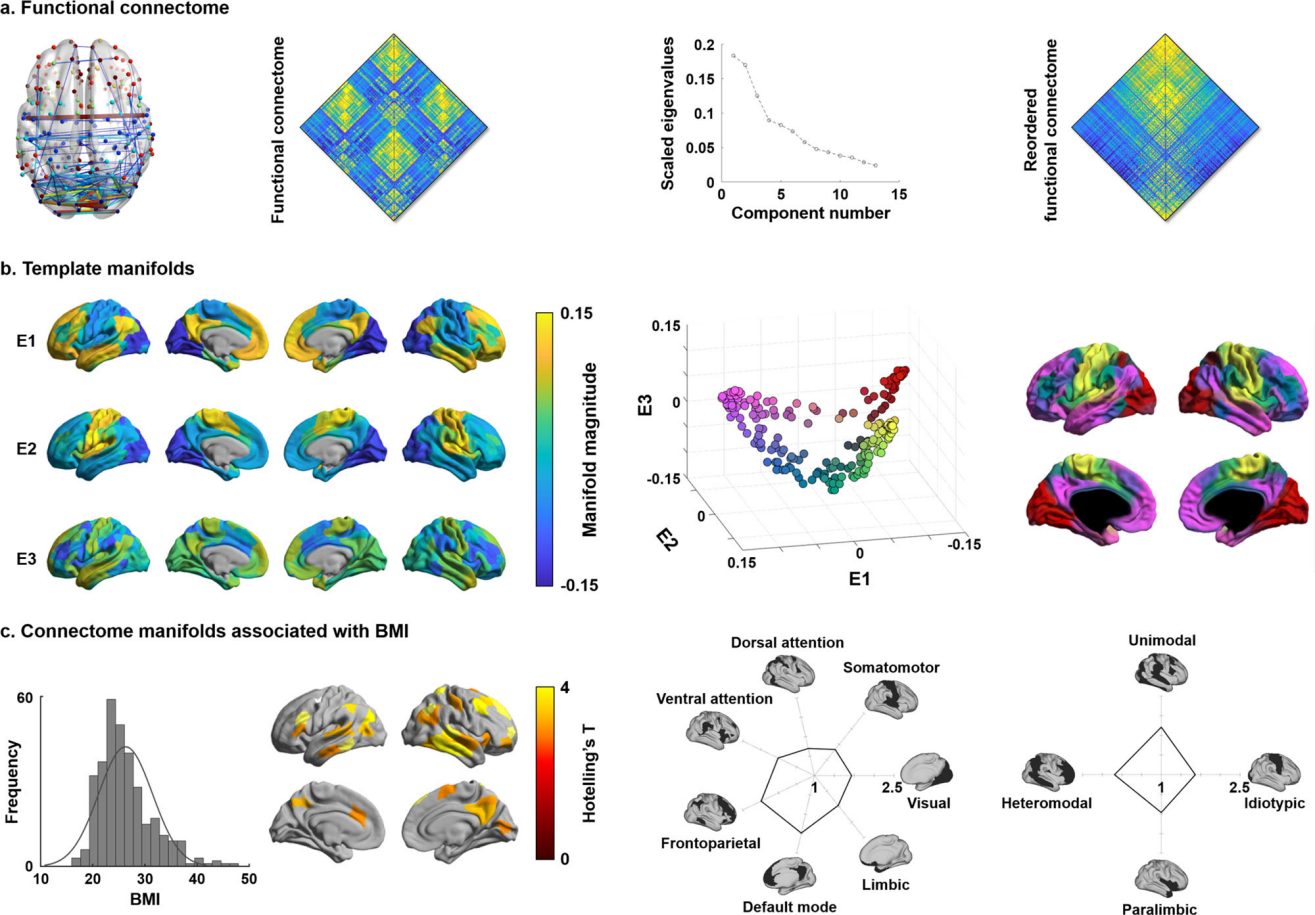
Functional connectome manifolds. **a** A group averaged functional connectome in graph (left) and matrix representation (middle left), and a scree plot describing connectome information across functional components (middle right). The reordered functional connectivity matrix according to the first eigenvector (i.e., E1) is shown on the right. **b** Template manifolds were built by three dominant eigenvectors (E1, E2, E3), based on the group averaged functional connectome (left). The scatter plot represents each brain region projected onto the three-dimensional manifold space with different colors (middle), also mapped to the cortical surface for visualization (right). **c** The distribution of BMI is reported on the left. Multivariate association highlighted regions showing significant associations between the three eigenvectors and inter-individual variations in BMI (middle left). Findings were corrected for multiple comparisons using a false discovery rate (FDR) < 0.05. Effects were stratified according to intrinsic functional communities^72^ (middle right) and levels of cortical hierarchy^46^ (right), and shown in the spider plots. Source data are provided in Supplementary Data 2.

We constructed functional connectomes in individual subjects based on the correlation analysis of rs-fMRI data and estimated functional manifolds^39^ using diffusion map embedding^69^ implemented in BrainSpace (https://github.com/MICA-MNI/BrainSpace; see “Methods”)^67^. The template manifold was estimated using the group averaged functional connectome, and we aligned individual manifolds to this template using Procrustes rotations^67,70^. We selected three eigenvectors (E1, E2, E3), explaining ~48% of information in the template affinity matrix (Fig. 1a, b). Each eigenvector (also referred to as *gradient*) represents an axis of spatial variation in the functional connectome. In accordance with prior findings in the HCP dataset^39,67^, the eigenvectors differentiated primary sensory areas from higher order transmodal areas (E1), visual from somatomotor cortices (E2), and the multiple demand network from the rest of the brain (E3).

Multivariate analysis associated the three eigenvectors with inter-individual differences in BMI, controlling for age and sex. Significant associations were identified in transmodal cortical areas (false discovery rate (FDR) < 0.05^71^; Fig. 1c). Stratifying the effects according to intrinsic functional communities^72^ and a model of cortical hierarchical laminar differentiation^46^, we revealed the highest effects in default mode and frontoparietal networks situated in both unimodal and heteromodal association cortices.

To express the multivariate pattern in a single scalar, we computed a compact *manifold eccentricity* metric for all participants^47,73^, which was calculated as the Euclidean distance between the center of template manifold and all data points (i.e., cortical regions) in manifold space (Supplementary Fig. 1a). The manifold eccentricity showed high value in somatosensory, lateral temporal, and medial prefrontal cortices, while frontoparietal and limbic regions showed low value. After controlling for age and sex, we could replicate an association between inter-individual variations in BMI and manifold eccentricity of the regions identified from the multivariate analysis (see Fig.1c; *p* < 0.001; non-parametric permutation tests; Supplementary Fig. 1b). To provide further topological context, we also calculated spatial associations between manifold eccentricity and graph-theoretical measures representing the integration/segregation of intrinsic functional communities. Specifically, we calculated within-module degree and participation coefficient^74,75^ based on an established intrinsic functional partitioning^72^ (Supplementary Fig. 2a–c). We found a significant positive correlation with within-module degree (*r* = 0.20, FDR < 0.001; non-parametric permutation tests followed by FDR across modular parameters), while participation coefficient was negatively correlated (*r* = −0.12, FDR = 0.02). Similar patterns were observed when defining modules using Louvain community detection algorithm^76^ (Supplementary Fig. 2d–f) or the Mesulam schema of cortical hierarchy and laminar differentiation^46^ (Supplementary Fig. 2g–i). These results indicate increased functional segregation of networks involved in transmodal areas in individuals with higher BMI.

### Associations to inter-individual variations in cortical morphology

**Fig. 2:**
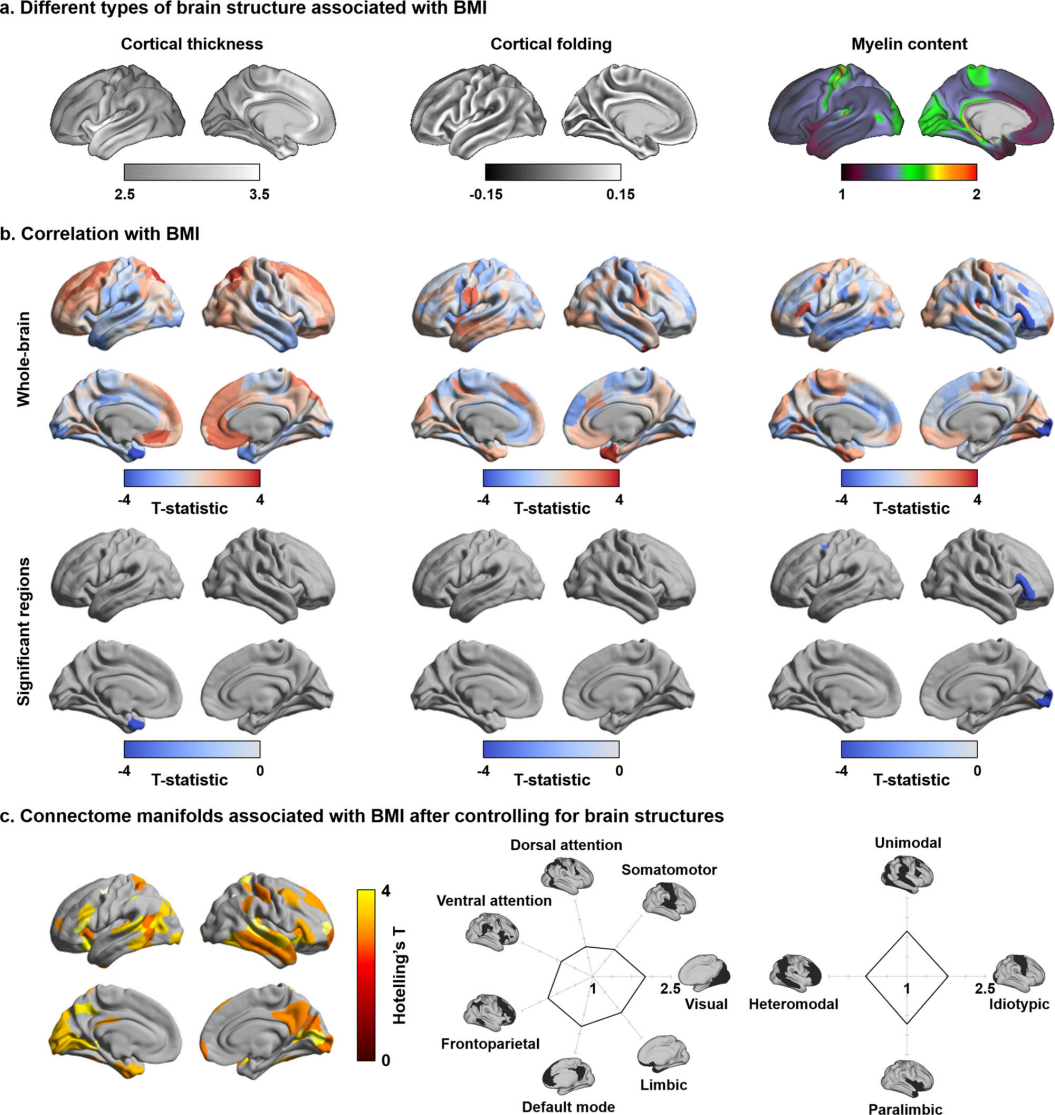
Effects of brain structures. **a** MRI measures of brain structure, including cortical morphology and intracortical microstructure were obtained in the same participants. **b** Linear correlations between inter-individual variations in BMI and these indices of brain structure. **c** Multivariate association of the three functional eigenvectors with BMI, after controlling for variations in brain structure. Source data are provided in Supplementary Data 2.

Previous studies have reported associations between individual differences in BMI and MRI measures of cortical thickness, cortical folding, and tissue microstructure^11,77–80^. Here, we explored whether functional connectome manifold findings were, in part, explainable by these underlying structural associations. We measured cortical morphology (cortical thickness and folding) and intracortical microstructure (the ratio between T1- and T2-weighted imaging contrast, a proxy for intracortical myelin) in the same subjects (Fig. 2a)^44,81,82^. Two analyses were performed. First, we correlated inter-individual differences in BMI with these indices of brain structure while controlling for age and sex. While cortical folding was not associated with BMI, a negative effect on cortical thickness was observed in the temporal pole (*r* = −0.21; FDR < 0.05), and we also found reductions in myelin proxies in occipital, central, and ventrolateral prefrontal regions in individuals with higher BMI (*r* = −0.35; FDR < 0.05) (Fig. 2b). Second, we repeated the analysis associating inter-individual differences in BMI to multivariate connectome manifolds (E1–E3) after controlling for the measures of brain structure. Findings were consistent with our main results, showing strong effects in default mode and frontoparietal networks (Fig. 2c). Collectively, these findings suggest that functional connectivity associations to inter-individual variations in BMI were robust above and beyond associations between BMI and measures of cortical morphology and microstructure.

### Transcriptomic association analysis

**Fig. 3:**
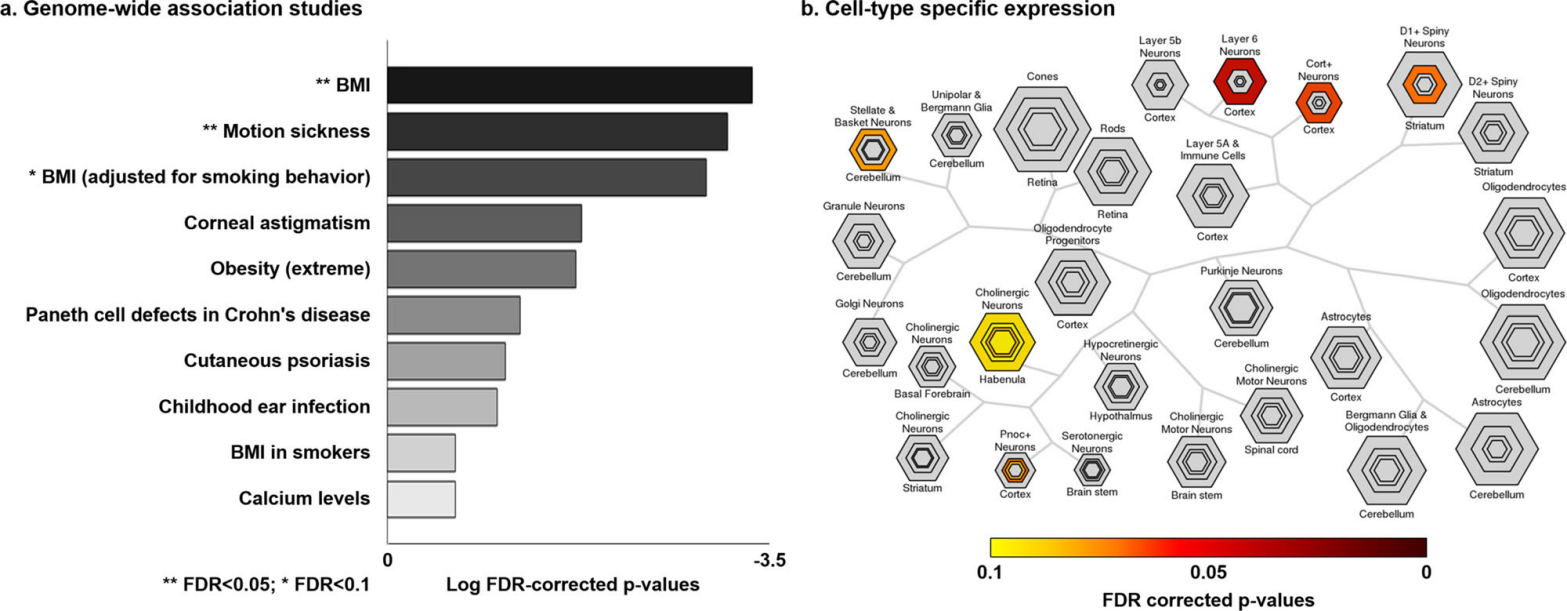
Transcriptomic analysis. **a** Genes were derived by associating map of BMI-related manifold changes and gene expression maps from Allen Brain Atlas. Top ten categories associated with gene expressions derived from genome-wide association studies. **b** Cell-type specific expression analysis identified candidate cell populations associated with genes expressed in the input spatial map (see Fig. 1c). The hexagon size represents the proportion of genes specifically expressed in a particular tissue. Varying stringencies for enrichment are represented by the size of hexagons going from least specific (outer hexagons) to most specific (center hexagons) (specificity index threshold (pSI) = 0.05, 0.01, 0.001, and 0.0001, respectively)^60^. Colors represent the FDR-corrected *p*-values. Source data are provided in Supplementary Data 2.

To explore neurobiological associations to our macroscale findings (see “Methods” for details), we correlated the spatial map of BMI-related functional manifold changes (see Fig. 1c) with cortical maps of *post-mortem* gene expression data obtained from the AIBS^54,83,84^. We repeated the correlation analysis with spin-rotated maps of BMI-related patterns 100 times to ensure that significantly associated genes (FDR < 0.05) were not selected by chance^85^. Among the significantly associated gene lists, only the genes consistently expressed across different donors (FDR < 0.05) (see “Methods”; Supplementary Data 1)^52^ were fed into the genome-wide association studies using Enrichr (https://amp.pharm.mssm.edu/Enrichr/)^59,61^. These findings pointed to the strongest effects for genes previously shown to be associated with BMI (FDR < 0.05; Fig. 3a). Furthermore, cell-type specific expression analysis (http://genetics.wustl.edu/jdlab/csea-tool-2/)^60^ suggested that genes associated with BMI-related functional manifold changes were enriched to cortical cells as well as those in striatum and cerebellum (FDR < 0.05; Fig. 3b) previously implicated in the regulation of food-related reward processing and appetite^86–89^. Specifically, genes were enriched for GABAergic cells of D1 medium spiny neurons in the striatum and stellate and basket cells in cerebellum, as well as in cortical neurons (FDR < 0.05).

### Sensitivity and replication experiments

A series of analyses evaluated robustness of our findings.

- *Head motion*. We repeated the multivariate analyses associating BMI with connectome manifolds after controlling for head motion, quantified as frame-wise displacement in rs-fMRI^90^. We observed overall increased effect sizes (+42%) in functional communities except for the frontoparietal network, which showed decreased effects (−14%) (Supplementary Fig. 3a).
- *Fluid intelligence, sleep quality, and blood pressure*. BMI has previously been related to fluid intelligence^11,91^, sleep quality^92,93^, as well as blood pressure^94,95^. These associations were confirmed in this dataset, showing low to moderate correlations between BMI and fluid intelligence (*r* = −0.17, FDR = 0.003; non-parametric permutation tests followed by FDR across covariates), quality of sleep (*r* = 0.13, FDR = 0.02), and blood pressure (*r* = 0.45/0.30, FDR < 0.001 for systolic/diastolic) after controlling for age and sex. Repeating the multivariate association analyses after additionally controlling for these factors, we obtained findings that were largely similar to our main results (Supplementary Fig. 3b–d).
- *Multivariate association with weight*. We additionally performed multivariate association analyses between weight and connectome manifolds with controlling for age and sex, as well as height. We found almost unchanged spatial patterns relative to our main findings (Supplementary Fig. 4a).
- *Group comparison*. Instead of carrying out a correlation analysis between functional manifolds and BMI, we also performed a multivariate group comparison to compare cortex-wide manifolds (E1–E3) between individuals with healthy weight (18.5 ≤ BMI < 25) and those with higher BMI (BMI ≥ 25). We observed virtually identical results to our main findings (Supplementary Fig. 4b).
- *Spatial scale*. As the main analysis was performed using the Schaefer atlas with 200 parcels^96^, we additionally evaluated the results at both coarser and finer parcellation schemes of 100, 300, and 400 parcels, respectively. Findings were consistent across all parcel resolutions, despite subtle variations in the exact pattern of findings (Supplementary Fig. 5).
- *Matrix thresholding*. While main findings were based on functional connectomes thresholded at a 10% density as in prior work^39,49,67^, we also repeated our analysis at 5, 15, and 20% densities (Supplementary Fig. 6). We found highly similar patterns at these densities (mean spatial correlation across manifold maps, *r* = 0.85).
- *Reproducibility in HCP validation dataset*. We repeated the main analyses in an independent dataset from the HCP S1200 (*n* = 74) and found largely consistent results, with frontoparietal and default mode networks showing high associations with BMI (Supplementary Fig. 7a–c).
- *Reproducibility in HCP dataset with bootstraps*. Among the whole HCP sample (*n* = 399), we associated eigenvectors and BMI using randomly selected 300 participants, and replicated the findings in the remaining 99 subjects, for a total of 1000 times (see “Methods”). We found that the results were consistent (Supplementary Fig. 7d, e).
- *Reproducibility in another dataset*. Using an independent dataset with different acquisition parameters (*n* = 36; see “Methods”), we replicated our main findings that the connectome manifolds in higher order heteromodal association areas are associated with BMI (Supplementary Fig. 8).
- *Association between BMI and graph-theoretical measures*. Correlating BMI to betweenness, eigenvector, and degree centrality^75,97,98^ with controlling for age and sex, we could not find significant associations (FDR > 0.8).

## Discussion

Human connectome organization can be conceptualized along multiple processing hierarchies^99^, which allow for integrative and segregated neural functions. Here, we assessed inter-individual differences in this architecture relative to phenotypic variations in BMI, a well-known predictor of health, wellbeing, and life expectancy^1–3^. Our approach leveraged techniques that decompose the whole-brain functional connectome into a set of eigenvectors differentiating macroscale systems in a gradual manner along the cortical surface. We observed that unimodal and heteromodal association areas are more differentiated in individuals with higher BMI, suggestive of a potentially disrupted segregation between different levels of the cortical hierarchy. Findings remained consistent when additionally controlling for inter-individual variations in MRI-based measures of cortical morphology and microstructure, suggesting that functional network associations with BMI existed above and beyond potential regional effects on local brain structure. Functional connectome changes were found in cortical territories known to harbor genes previously implicated in BMI variations, as well as those involved in cortical, striatal, and cerebellar cells. These findings suggest functional network substrates of inter-individual variations in BMI that may ultimately reflect macroscale effects of cellular-genetic associations to BMI.

Manifold learning techniques were utilized to represent macroscale functional connectomes through a series of lower-dimensional eigenvectors. Studying the HCP cohort, we identified three eigenvectors that each described a spatial gradient in cortico-cortical functional differentiation and that collectively explained approximately 50% of connectivity information. The overall pattern of gradients was in agreement with earlier studies in the same dataset^39,67^, with a principal gradient differentiating sensorimotor and transmodal systems, a second gradient differentiating sensorimotor and visual networks, and a third gradient being sensitive to a differentiation of the multiple demand network from the rest of the brain^39,100^. Notably, associating inter-individual differences in BMI with manifold organization, we observed a marked modulation of functional gradients by inter-individual differences in BMI. Findings were particularly visible in association cortices that encompass integrative default mode and frontoparietal networks. Further contextualization with manifold eccentricity and graph theoretical parameters indicated segregation of association cortices in individuals with higher BMI. Prior fMRI studies reported atypical intrinsic functional connectivity in individuals with obesity, at both nodal and global network levels, relative to individuals with healthy weight^30,34,35,101–103^. Our findings complement these previous reports focusing on the analysis of connectivity patterns of specific areas^30–32,35^ alongside prior graph-theoretical analyses^30,34,103^ in the context of person-to-person variations in BMI. Prior functional connectivity studies found that individuals with obesity showed increased connectivity in nodes belonging to frontoparietal and default mode networks^30,35,101^. These findings also parallel work showing positive associations between overall network connectivity and BMI, again frequently observed in networks situated in transmodal association cortex^34,37,103^. Longitudinal evidence also points to an association between BMI changes and connectivity of reward and frontoparietal networks, both when following healthy individuals over time^104^ and secondary to repetitive transcranial magnetic stimulation targeting the dorsolateral prefrontal cortex^105^. Beyond work focusing on associations between inter-individual differences in BMI and localized connectivity patterns, a more recent study reported increased modular segregation of functional networks as BMI increases^106^. A more segregated network organization has previously been reported in several psychiatric and neurological diseases, including attention deficit hyperactivity disorder^107–111^, Alzheimer’s disease^111–114^, and impulsivity^115^. These studies noted that increased segregation might reduce global network efficiency and delay information transfer between nodes^116–118^, potentially contributing to cognitive decline^111,119^. Based on these studies, the observed alterations in unimodal and heteromodal association cortices in individuals with higher BMI in our work could reflect disruptions in feedforward and feedback processing, and indicate atypical cognitive flexibility^7,8,11,15,34,120–123^.

While exploring associations between BMI variations and functional connectome organization may illustrate brain substrates of obesity^7,11,17,18,21,26,30,36,124^, BMI measure is not per se an indication of body fat distribution^125^. Central obesity measures, such as waist circumference and waist-to-hip ratio are alternative proxies for obesity, in particular for abdominal obesity. Indeed, BMI is strongly associated with these central obesity measures^125^ and shows similar or better reproducibility of predicting cardiovascular disease risk^126^. Although the interpretation of BMI should be carefully discussed, BMI is a widely adopted index of obesity in the clinics and has been used to examine brain substrates associated to BMI variations^7,8,13,32,34,104,105,122^. Of note, our findings were largely consistent when incorporating a range of potential confounds, including fluid intelligence, sleep quality, and blood pressure. Moreover, we could observe similar patterns in an initially held out HCP subsample, as well as in a completely different dataset, supporting that our findings appear overall robust. It should be noted that, however, head motion was found to be related to BMI, in a rather complex way. Indeed, additionally controlling for head motion parameters in our statistical models increased the effect sizes in several networks but reduced effect sizes in the frontoparietal network. A prior study showed weight loss is related to reduced head motion, suggesting a need to assess head motion effects in obesity neuroimaging studies^127^. Further work is needed to identify mediating factors between head motion and body weight, and to determine how to optimally include head motion parameters in BMI/obesity neuroimaging.

In addition to its conceptual alignment with established models of cortical hierarchical organization^39,46^, the manifold framework allowed for the projection of connectome-derived findings back to cortical surfaces. In our analyses, we could thus integrate functional findings with morphological and microstructural measures in the same participants. Previous studies have explored morphological substrates of BMI variations, reporting cortical thinning in lateral prefrontal, entorhinal, and parahippocampal regions as BMI increases, indicating that overweight and obese people have reduced cortical thickness compared to people with a normal body weight^11,78,80,128–130^. A recent multi-site study confirmed that high BMI (≥ 30) relates to reduced thickness in temporal and frontal cortices^131^. In our study, we observed diffuse tendencies for decreased cortical thickness in individuals with higher BMI, with significant peak effects in temporopolar cortices. Findings were complemented by microstructural associations in primary sensory and ventrolateral prefrontal cortices, potentially indicative of myelin anomalies in individuals with high BMI that have already been suggested based on different methodologies^132–134^. Notably, associations between BMI and functional connectome organization were virtually unchanged when controlling for MRI-derived indices of morphology and microstructure. These findings indicate that the functional connectome reorganization situated in higher order brain regions likely occurred above and beyond these underlying structural variations.

In addition to MRI-based analyses of regional morphology and microstructure, we performed a transcriptomic association analysis based on *post-mortem* gene expression maps provided by the Allen Brain Atlas. Although such transcriptomic associations were established through a different and small dataset that is not necessarily representative of the HCP sample, equivalent approaches have been increasingly adopted in neuroimaging research to identify genes whose expression patterns covary with macroscopic findings^45,50,62–66^. In our work, spatial association analyses pointed to specific gene sets, which were cross-referenced with previously reported genome-wide association studies. This analysis demonstrated that the topography of functional connectome manifold changes seen in the current study co-localized with the expression pattern of genes previously implicated in BMI variations through genome-wide association studies. These findings could thus indicate that the functional connectome associations with BMI may ultimately reflect macroscale effects of genetically mediated processes, a finding to be further validated with direct imaging-genetics approaches. Additional gene set enrichment analyses suggested that the identified genes are mainly expressed by cortical neurons, together with cells in the cerebellum, as well as D1 medium spiny neurons in the striatum. Although these associations are indirect and based on different samples, they may extend and recapitulate computational theories on circuit mechanisms contributing to BMI, and notably point to an atypical organization of dopaminergic circuits involving mesolimbic and cortical control systems in high BMI/obesity^86–89^.

In sum, our study identified functional connectome substrates of inter-individual BMI variations in healthy young adults based on connectome manifold learning. Our findings point to altered modular and hierarchical organization of the brain, specifically between unimodal and heteromodal association cortices. These findings were robust with respect to several confounds and variations in cortical morphology, and could be replicated in several datasets. Transcriptomic decoding suggested that these patterns were spatially associated with the expression of genes previously implicated in BMI variations as well, potentially related to cortical-subcortical dopamine signaling pathways. Our findings, thus, suggest coupled macroscale and molecular substrates of BMI variations in the adult human brain.

## Methods

### Participants

We obtained the minimally processed imaging and phenotypic data from the S900 release of HCP^68^. We excluded participants who are genetically related (i.e., twin pairs; *n* = 461), and who did not complete full imaging data with acceptable image quality (i.e., less than one T1- and T2-weighted and four sessions of rs-fMRI; *n* = 169), resulting in a total of 325 participants (mean ± SD age = 28.56 ± 3.74 years; 55% female). The mean BMI of the participants was 26.30 kg/m^2^ with an SD of 5.16 (range = 16.65–47.76 kg/m^2^), and the proportion of underweight (BMI < 18.5 kg/m^2^), healthy weight (18.5 ≤ BMI < 25 kg/m^2^), overweight (25 ≤ BMI < 30), and obesity (BMI ≥ 30) was 6:143:113:63. We selected additional data from the S1200 release of HCP to replicate the findings (see “Sensitivity and reproducibility analyses” section). Identical exclusion criteria were applied (twin pairs *n* = 144; without full imaging *n* = 18). A total of 74 participants (mean ± SD age = 28.08 ± 3.90 years; 34% female; mean ± SD BMI = 26.17 ± 4.39 kg/m^2^, range 18.89–39.47 kg/m^2^) were enrolled, and the ratio of healthy weight, overweight, and obesity was 30:29:15. All MRI data used in this study were publicly available and anonymized. Participant recruitment procedures and informed consent forms, including consent to share de-identified data, were previously approved by the Washington University Institutional Review Board as part of the HCP. In addition, we analyzed an independent dataset from an independent site (St. Vincent’s Hospital (SVH): *n* = 36; mean ± SD age = 38.78 ± 10.52 years; 47% female; mean ± SD BMI = 29.38 ± 6.29 kg/m^2^, range 23.15–57.13 kg/m^2^). Data collection and usage were approved from the Institutional Review Boards of the Catholic University of Korea (no. XC15DIMI0012, approved March 2015), and written and informed consent was obtained from all participants.

### MRI acquisition

- *HCP:* HCP imaging data were obtained on a Siemens Skyra 3T at Washington University. The T1-weighted images were acquired using a magnetization-prepared rapid gradient echo (MPRAGE) sequence (repetition time (TR) = 2400 ms; echo time (TE) = 2.14 ms; field of view (FOV) = 224 × 224 mm^2^; voxel size = 0.7 mm^2^; and number of slices = 256). The T2-SPACE sequence was used for scanning T2-weighted structural data, and the acquisition parameters were the same as the T1-weighted data except for TR (3200 ms) and TE (565 ms). The rs-fMRI data were collected using a gradient-echo EPI sequence (TR = 720 ms; TE = 33.1 ms; FOV = 208 × 180 mm^2^; voxel size = 2 mm^3^; number of slices = 72; and number of volumes = 1200). During the rs-fMRI scan, participants were instructed to keep their eyes open looking at a fixation cross. Two sessions of rs-fMRI data were acquired; each of them contained data of left-to-right and right-to-left phase-encoded directions, providing up to four time series per participant.
- *SVH:* The SVH imaging data were scanned using a Siemens Magnetom 3T scanner equipped with a 32-channel head coil. The T1-weighted images were acquired using a MPRAGE sequence (TR = 1900 ms; TE = 2.49 ms; FOV = 250 × 250 mm^2^; voxel size = 1 mm^3^; and number of slices = 160). The rs-fMRI data were collected using a gradient-echo EPI sequence (TR = 2490 ms; TE = 30 ms; FOV = 220 × 220 mm^2^; voxel size = 3.4 × 3.4 × 3 mm^3^; number of slices = 36; and number of volumes = 150).

### Data preprocessing

- *HCP:* HCP data were minimally preprocessed using FSL, FreeSurfer, and Workbench^135–137^. Structural MRI data were corrected for gradient nonlinearity and b0 distortions, and co-registration was performed between the T1- and T2-weighted data using a rigid-body transformation. Bias field was adjusted using the inverse intensities from the T1- and T2-weighting. Processed data were nonlinearly registered to MNI152 space, and white and pial surfaces were generated by following the boundaries between different tissues^138–140^. The midthickness surface was generated by averaging white and pial surfaces, and it was used to generate the inflated surface. The spherical surface was registered to the Conte69 template with 164 k vertices^141^ using MSMAll^142^ and downsampled to a 32 k vertex mesh. The rs-fMRI data were preprocessed as follows: first, EPI distortions and head motion were corrected, and data were registered to the T1-weighted data and subsequently to MNI152 space. Magnetic field bias correction, skull removal, and intensity normalization were performed. Noise components attributed to head movement, white matter, cardiac pulsation, arterial, and large vein related contributions were removed using FMRIB’s ICA-based X-noiseifier (ICA-FIX)^143^. Preprocessed time series were mapped to the standard grayordinate space, with a cortical ribbon-constrained volume-to-surface mapping algorithm. The total mean of the time series of each left-to-right/right-to-left phase-encoded data was subtracted to adjust the discontinuity between the two datasets and they were concatenated to form a single time series data.
- *SVH:* Data were processed using the fusion of the neuroimaging preprocessing (FuNP) pipeline integrating AFNI, FSL, FreeSurfer, and ANTs^135–137,144–146^. T1-weighted data were processed using equivalent procedures as for HCP. The rs-fMRI preprocessing removed the first 10 s (5 volumes) to allow for magnetic field saturation. Head motion and slice timing were corrected, and volumes with frame-wise displacement >0.5 mm removed. After removing non-brain tissues, intensity was normalized. Effects of head motion, white matter, cerebrospinal fluid, cardiac pulsation, and arterial and large vein related contributions were removed using ICA-FIX^143^. Registration from fMRI onto the T1-weighted data and subsequently to the ICBM-MNI152 3 mm^3^ standard space was performed. Data were band-pass filtered to within 0.009 and 0.08 Hz, and we applied spatial smoothing with a full width at half maximum of 5 mm. Processed fMRI data were mapped to the cortical surface with a cortical ribbon-constrained volume-to-surface mapping algorithm.

### Low dimensional functional manifold identification

We generated functional connectomes by computing linear correlations of the time series between two different brain regions, using the Schaefer 7-network based atlas with 200 parcels^96^. Correlation coefficients underwent Fisher’s r-to-z transformations to render data more normally distributed^62^. Cortex-wide functional manifolds (i.e., the principal eigenvectors explaining spatial shifts in the functional connectome) were estimated using BrainSpace (https://github.com/MICA-MNI/BrainSpace)^67^. A template manifold was estimated from the group average functional connectome (Fig. 1a). An affinity matrix, capturing the similarity of connections among different brain regions, was constructed using a normalized angle kernel with a connection density of 10%. We generated the connectome manifolds (Fig. 1b) using diffusion map embedding^69^, a robust and computationally efficient non-linear technique^147,148^. It is controlled by two parameters α and t, where α controls the influence of the density of sampling points on the manifold (*α* = 0, maximal influence; *α* = 1, no influence) and t scales eigenvalues of the diffusion operator. As in prior applications^39,44,49,67^, we set *α* = 0.5 and *t* = 0 to retain the global relations between data points in the embedded space. In this new manifold, interconnected brain regions are closely located, and the regions with weak inter-connectivity are located farther apart. Individual-level manifolds were estimated and aligned to the template manifold via Procrustes alignment^67,70^.

### Macroscale connectome associated with body mass index

We performed multivariate association analysis between BMI and the first three eigenvectors, which explained approximately 50% in connectome information, with the model controlling for age and sex. We utilized SurfStat (http://www.math.mcgill.ca/keith/surfstat/) to fit linear models to multivariate data (of the form number of subjects × number of brain regions × number of eigenvectors) as follows:

1 $${\rm{Y}}={\rm{b}}{0}+{\rm{b}}{1}\cdot {\rm{Age}}+{\rm{b2}}\cdot {\rm{Sex}}+{\rm{b}}{3}\cdot {\rm{BMI}}$$

The inference was based on Hotelling’s *t*-test in each parcel, and multiple comparisons were corrected for using the FDR procedure^71^. Our work focused on BMI. Although BMI measure may be limited in assessing the distribution of body fat, it is widely adopted for indexing obesity in the clinics and for exploring obesity-related associations to large-scale brain network organization^7,8,13,32,34,104,105,122^. We summarized multivariate statistics within established resting-state functional communities^72^ and with respect to levels of cortical hierarchy and laminar differentiation (Fig. 1c)^46^. The latter model, initially formulated in non-human primates, has recently been extended to humans based on in vivo functional connectome analysis as well as the mapping of microstructural gradients from *post-mortem* histology and myelin-sensitive 3T MRI^39,50^. Refinements of these models are to be expected by ongoing developments in high and ultra-high field scanning, which will offer higher resolution approximations of depth-specific cortical microstructure^149–152^.

We furthermore simplified the multivariate manifolds into a single scalar, manifold eccentricity, by calculating the Euclidean distance between the center of template manifold and all data points (i.e., brain regions) in the manifold space for each individual (Supplementary Fig. 1a)^47,73^. Manifold eccentricity has previously been related to clustering and path length, underscoring its capacity to index network segregation and integration^100^. We linearly correlated BMI and manifold eccentricity in regions identified by the multivariate analysis, controlling for age and sex (Supplementary Fig. 1b). Significance was assessed using 5,000 permutation tests. A null distribution was constructed and the real correlation strength was deemed significant if it did not belong to the 95% of the distribution (two-tailed *p* < 0.05). To assess how the modular architecture changes according to connectome manifolds, we calculated linear correlations between manifold eccentricity and modular measures of within-module degree and participation coefficient^74,75^ in the regions identified by the multivariate analysis (Supplementary Fig. 2). Modules were defined using established intrinsic functional communities^72^, a Louvain community detection algorithm^76^, and a schema of cortical hierarchy^46^. Within-module degree is the degree centrality within a module, indicating the intra-modular connections, while participation coefficient represents inter-modular connections^74,75^. In other words, high within-module degree represents that a given node has the property of being a hub node within a given module. In contrast, high participation coefficient indicates the node has edges distributed equally to other modules. The significance of the associations between manifold eccentricity and modular measures were assessed using 5000 permutation tests by randomly shuffling subjects. Multiple comparisons across different modular parameters were corrected using FDR^71^.

### Associations to brain structure

To assess morphological and microstructural associations (Fig. 2a), we first correlated BMI with MRI-based measures of cortical morphology, i.e., cortical thickness and cortical folding, and in vivo proxies of intracortical microstructure, i.e., the ratio of the T1-weighted and T2-weighted imaging contrast in voxels between the white and pial surfaces^44,81,82^, with controlling for age and sex (Fig. 2b). We repeated the association analysis between BMI and manifold changes, after controlling for these regional structural indices (Fig. 2c). Multiple comparisons were corrected for using FDR^71^.

### Transcriptomic association analysis

Transcriptomic association analysis explored co-varying neuromolecular properties of our functional connectome manifold findings (Fig. 3)^52,59–61,83,84^. Specifically, we correlated the t-statistical map of manifold changes associated with BMI with *post-mortem* gene expression maps from the AIBS using the Neurovault gene decoding tool^83,84^. Neurovault implements mixed-effect analysis to estimate associations between the input t-statistic map and the genes of AIBS donor brains yielding the gene symbols associated with the input t-statistic map. To validate whether the gene symbols passing FDR < 0.05 were derived by chance or not, we repeated the correlation analysis using 100 randomly rotated cortical maps of the multivariate association analysis^85^. We, thus, constructed a null distribution of spatial correlations between the expression patterns of the identified gene list and randomly rotated maps. The actual correlation t-statistic was placed into this null distribution to assess significance, and findings were again FDR-corrected^71^. Selected gene symbols were further tested whether they are consistently expressed across donors using abagen (https://github.com/rmarkello/abagen)^52,84,153^. For each gene, we estimated the whole-brain expression map for each donor, and correlated maps between all pairs of donors. It should be noted that in four of six donors, the right hemisphere gene expression was obtained from mirroring left hemisphere gene expression. Genes showing consistent a whole-brain expression pattern across donors (FDR < 0.05) were compared with genes extracted from genome-wide association studies using Enrichr (https://amp.pharm.mssm.edu/Enrichr/)^59,61^. Then we fed the consistent genes into the cell-type-specific expression analysis (http://genetics.wustl.edu/jdlab/csea-tool-2/) to identify candidate cell populations likely to be associated with input gene lists^60^. Significances were assessed using a z-score modification of Fisher’s exact test and FDR correction.

### Sensitivity and reproducibility analyses

- *Head motion*. To assess the effects of head motion on connectome manifolds, we repeated the multivariate association analysis while controlling for age and sex, as well as head motion. The latter was calculated from the frame-wise displacement during the rs-fMRI scan (Supplementary Fig. 3a).
- *Fluid intelligence, sleep quality, and blood pressure*. BMI has been related to fluid intelligence^11,91^, sleep quality^92,93^, and blood pressure^94,95^. To assess the relationship between BMI and these factors, we obtained fluid intelligence score from the Penn Progressive Matrices^154^ and quality of sleep from the Pittsburgh Sleep Quality Index^155–157^, as well as systolic and diastolic blood pressure measures. We repeated multivariate analyses to associate connectome manifolds and BMI with controlling for age and sex, as well as each of these factors (Supplementary Fig. 3b–d).
- *Multivariate association with weight*. We performed multivariate analyses to associate connectome manifolds with weight after controlling for age, sex, and height to cross-validate our main findings (Supplementary Fig. 4a).
- *Group comparison*. We compared connectome manifolds spanned by E1–E3 between individuals with healthy weight (18.5 ≤ BMI < 25) to those being overweight (BMI ≥ 25), controlling for age and sex, to assess whether the findings from multivariate association to BMI are similar to those from multivariate group comparison (Supplementary Fig. 4b). Six underweight (BMI < 18.5) individuals were excluded.
- *Spatial scale*. To evaluate the impact of spatial scale, we repeated our analyses across different scales of the Schaefer atlas (i.e., 100, 300, or 400 regions) (Supplementary Fig. 5)^96^.
- *Matrix thresholding*. We repeated manifold estimation using functional connectomes with different levels of density from 5 to 20% with an interval of 5% (Supplementary Fig. 6).
- *Reproducibility in HCP validation dataset*. We performed the same analyses using the validation dataset obtained from the S1200 release of the HCP to replicate our findings (*n* = 74) (Supplementary Fig. 7a–c).
- *Reproducibility in HCP dataset with bootstraps*. We performed the same analyses using the dataset combined HCP S900 with S1200 release (*n* = 399). We randomly selected 300 subjects and associated the estimated eigenvectors with BMI, with controlling for age and sex. We repeated this procedure 1,000 times to avoid subject selection bias. For each iteration, we also performed the association analysis using the remained 99 subjects to assess robustness (Supplementary Fig. 7d, e).
- *Reproducibility in another dataset*. We replicated our findings using the independent SVH dataset (*n* = 36) (Supplementary Fig. 8).
- *Association between BMI and graph-theoretical measures*. To compare the sensitivity of manifold learning techniques to conventional approaches, we calculated graph-theoretical measures of betweenness, eigenvector, and degree centrality and assessed associations with BMI^75,97,98^. Betweenness centrality is the number of weighted shortest paths between any combinations of nodes that run through that node, eigenvector centrality measures the influence of a node in the whole network, and degree centrality is the sum of edge weights connected to a given node. We associated BMI with these graph measures while controlling for age and sex.

### Statistics and reproducibility

We associated connectome manifolds and BMI using multivariate linear regression models while controlling for age and sex. We calculated Hotelling’s *t*-statistics and the significance was corrected for multiple comparisons across the brain regions using FDR^71^. The association between BMI and manifold eccentricity, controlling for age and sex, was assessed using 5000 permutation tests by randomly shuffling subjects, and significance was determined based on two-tailed *p* < 0.05. We assessed the association between manifold eccentricity and modular parameters based on 5000 permutation tests followed by FDR^71^. The morphological associations to BMI were assessed using linear correlations, after controlling for age and sex. These findings were also corrected for multiple comparisons based on FDR^71^. Transcriptomic associations between t-statistics of manifold changes and maps of gene expressions were performed using mixed-effect analysis, where the significance was determined by 100 spin tests followed by FDR^71,85^. The significance of the cell-type specific expression analysis was assessed using a z-score modification of Fisher’s exact test and FDR correction. The reproducibility was assessed in the HCP S1200 dataset through 1000 bootstraps that randomly selected 300 subjects from the initial 399 participants, as well as using an independent SVH dataset (*n* = 36).

### Reporting summary

Further information on research design is available in the Nature Research Reporting Summary linked to this article.

## Supplementary information

Supplementary Information

Description of Supplementary Files

Supplementary Data 1

Supplementary Data 2

Reporting Summary

## Data Availability

The imaging and phenotypic data were provided, in part, by the Human Connectome Project, WU-Minn Consortium (https://www.humanconnectome.org/) and they are available after approval. Data from St. Vincent’s Hospital are not publicly available due to IRB restrictions. The subsets of data from these databases that were used in the present work are available from the authors upon request. Source data are provided with this paper as Supplementary Data 2.

## Code availability

The codes for data preprocessing are available at https://github.com/Washington-University/HCPpipelines and https://gitlab.com/by9433/funp, for connectome manifold generation are at https://github.com/MICA-MNI/BrainSpace, for manifold eccentric calculation are at https://github.com/MICA-MNI/micaopen/tree/master/manifold_features, and for graph measure calculation are at https://sites.google.com/site/bctnet. Transcriptomic association analyses were conducted using NeuroVault (https://neurovault.org), cell-type-specific expression analysis (http://genetics.wustl.edu/jdlab/csea-tool-2/)^60^, and abagen tools (https://github.com/rmarkello/abagen)^52,84,153^.
