# Supplementary material for: Inter-individual body mass variations relate to fractionated functional brain hierarchies
Source: Commun Biol. 2021 Jun 14;4:735. doi: 10.1038/s42003-021-02268-x (PMC8203627; doi:10.1038/s42003-021-02268-x)
Supplement: Supplementary file 1 — Supplementary Information [file 42003_2021_2268_MOESM1_ESM.pdf]

SUPPLEMENTARY INFORMATION

INTER-INDIVIDUAL BODY MASS VARIATIONS RELATE TO FRACTIONATED FUNCTIONAL BRAIN HIERARCHIES

Bo-yong Park[1,2*], Hyunjin Park[3,4], Filip Morys[1], Mansu Kim[5], Kyoungseob Byeon[4,6], Hyebin Lee[4,6], Se-Hong Kim[7], Sofie Valk[8], Alain Dagher[1], Boris C. Bernhardt[1*]

[1]McConnell Brain Imaging Centre, Montreal Neurological Institute and Hospital, McGill University, Montreal, QC, Canada; [2]Department of Data Science, Inha University, Incheon, Republic of Korea; [3]School of Electronic and Electrical Engineering, Sungkyunkwan University, Suwon, Republic of Korea; [4]Center for Neuroscience Imaging Research, Institute for Basic Science, Suwon, Republic of Korea; [5]Department of Biostatistics, Epidemiology, and Informatics, University of Pennsylvania, Philadelphia, United States of America; [6]Department of Electrical and Computer Engineering, Sungkyunkwan University, Suwon, Republic of Korea; [7]Department of Family Medicine, St. Vincent's Hospital, Catholic University College of Medicine, Suwon, Republic of Korea; [8]Otto Hahn Research Group for Cognitive Neurogenetics, Max Planck Institute for Cognitive and Brain Sciences, Leipzig, Germany

***Corresponding Authors:**

Bo-yong Park, PhD
Department of Data Science
Inha University
Incheon, Republic of Korea
Phone: +82-32-860-9427
Email: boyong.park@inha.ac.kr

Boris C. Bernhardt, PhD
Multimodal Imaging and Connectome Analysis Lab
McConnell Brain Imaging Centre
Montreal Neurological Institute and Hospital
McGill University
Montreal, Quebec, Canada
Phone: +1-514-398-3579
Email: boris.bernhardt@mcgill.ca

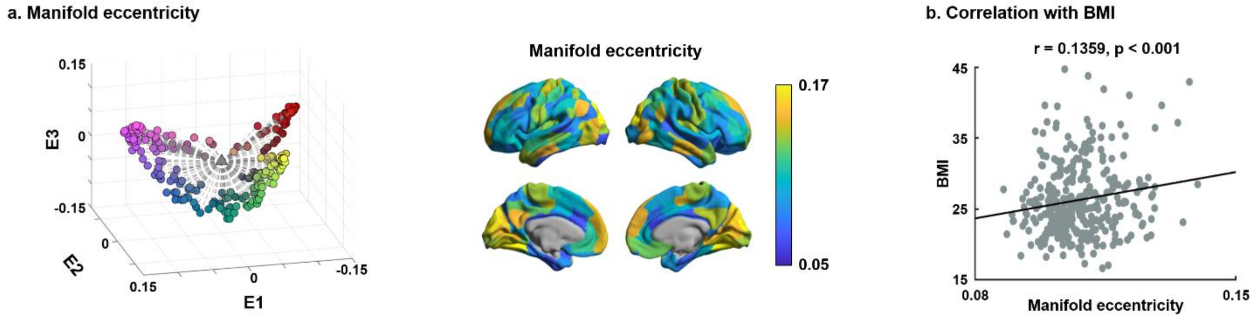

Supplementary Fig. 1: Association between BMI and manifold eccentricity.

(**a**) Manifold eccentricity measured as a Euclidean distance between the center of the template manifold and each data point. (**b**) Linear correlation between BMI and manifold eccentricity. *Abbreviation:* BMI, body mass index.

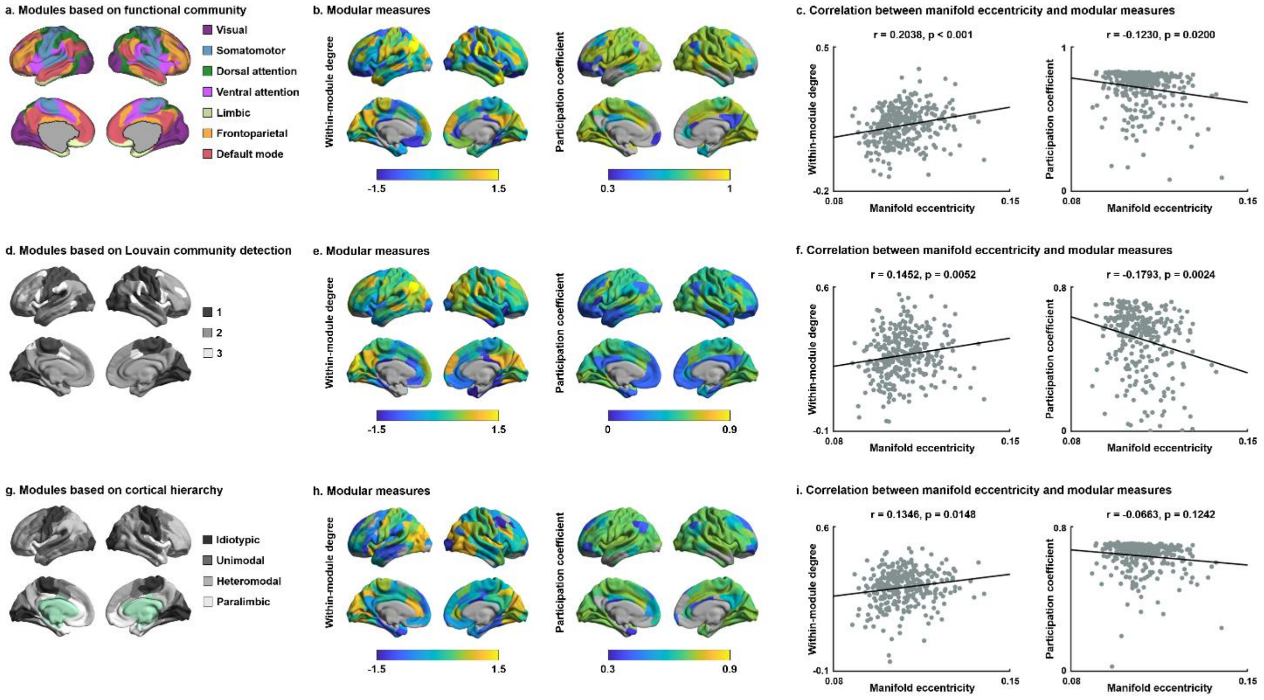

Supplementary Fig. 2: Manifold eccentricity and modular measures.

(a) Functional communities based on intrinsic functional communities [Supplementary 1]. **(b)** Distribution of within-module degree (left) and participation coefficient (right) in the whole-brain. **(c)** Linear correlation between manifold eccentricity and modular measures in the identified regions from the multivariate analysis (see *Fig. 1c*). (**d–f**) Results based on the modules defined using Louvain community detection [Supplementary 2] and (**g–i**) cortical hierarchy [Supplementary 3].

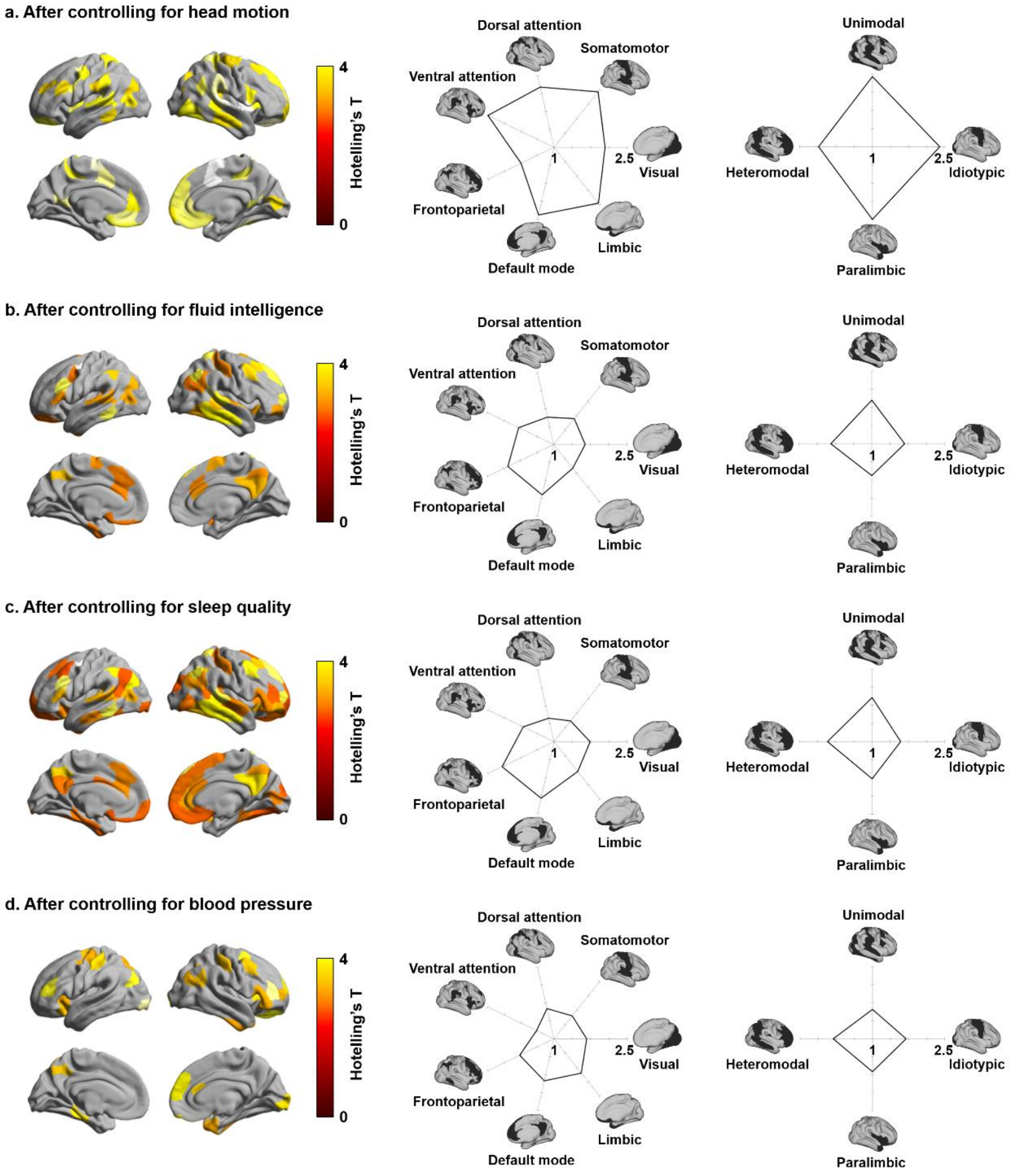

Supplementary Fig. 3: Multivariate association analysis between BMI and connectome manifolds after controlling for covariates.

(a) Results controlling for head motion, **(b)** fluid intelligence, **(c)** sleep quality, and **(d)** blood pressure.

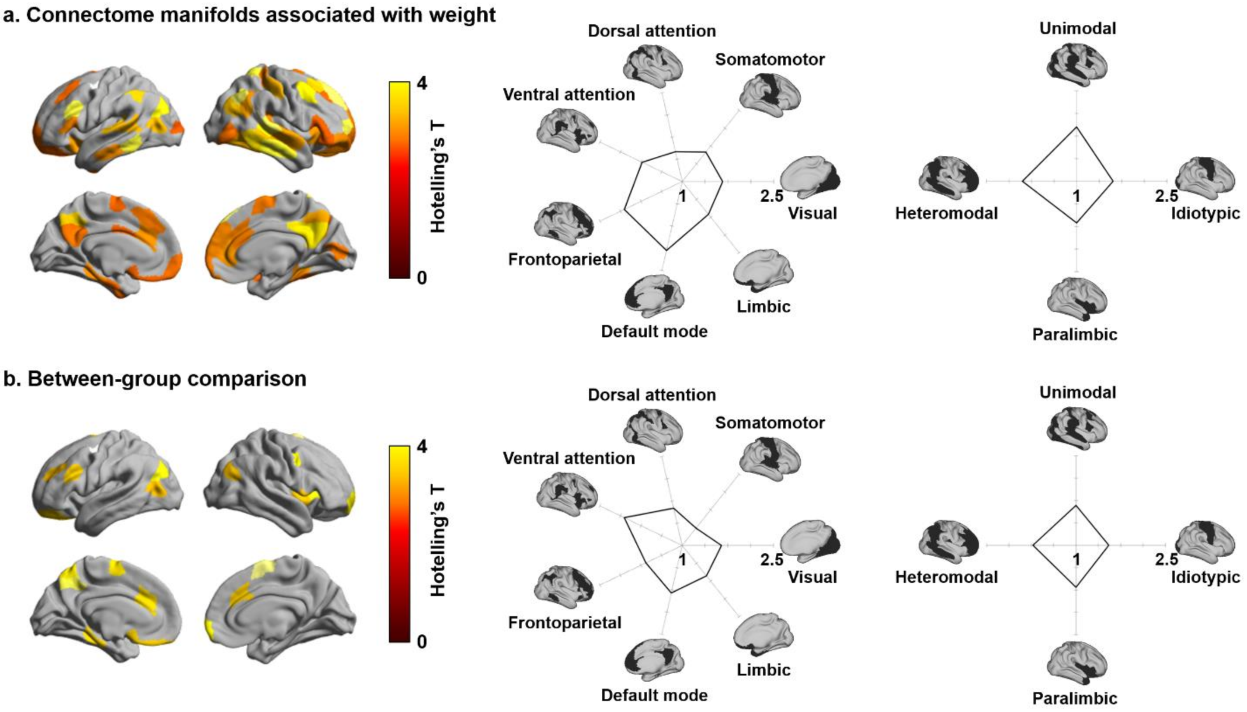

Supplementary Fig. 4: Additional multivariate analyses.

(a) The t-statistics of the identified regions that showed significant multivariate association analysis between connectome manifolds and weight. **(b)** Those for between-group differences in connectome manifolds between individuals with healthy (18.5 ≤ BMI < 25) and non-healthy weight (BMI ≥ 25).

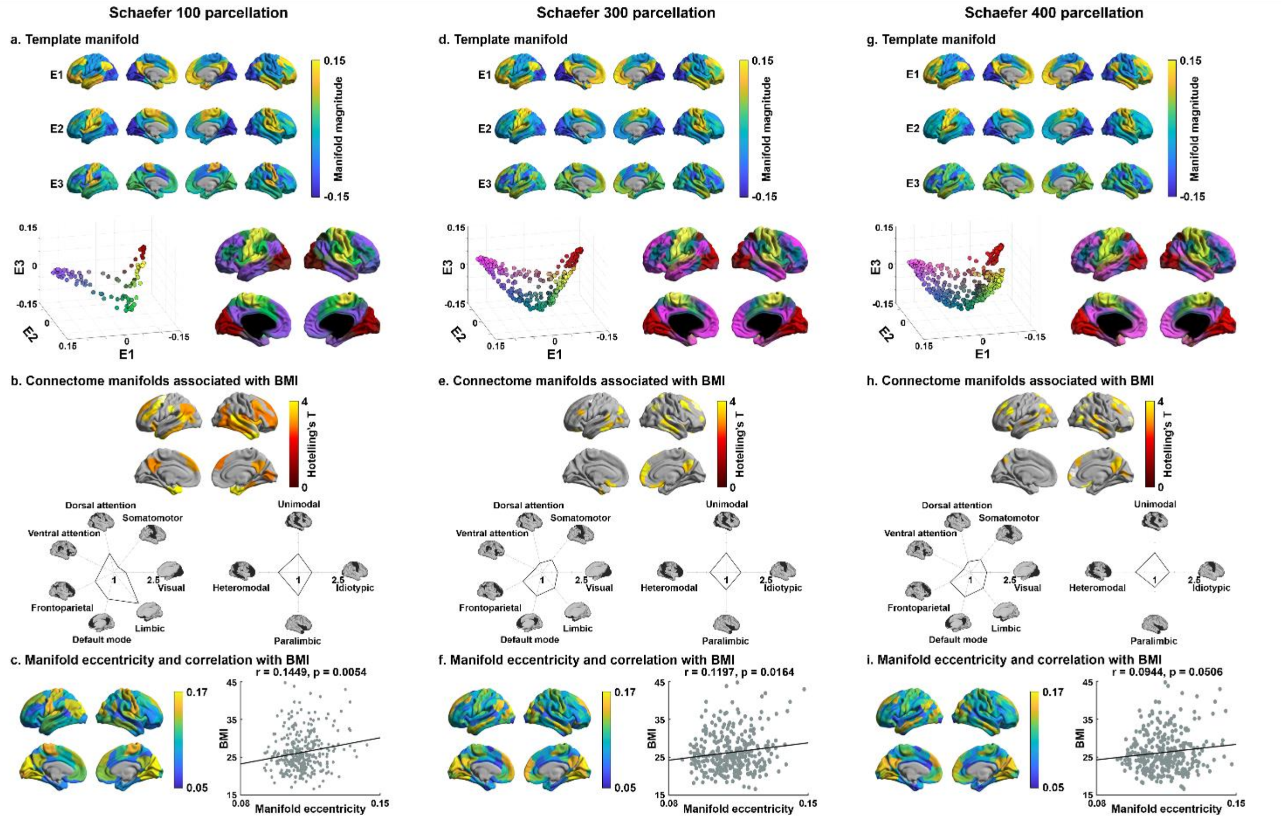

Supplementary Fig. 5: Results based on the different Schaefer parcellation schemes Supplementary 4**.**

(a)–(c) Schaefer 100, **(d)–(f)** 300, and **(g)–(i)** 400 atlases. For details, see *Fig. 1 and S1*. *Abbreviations:* BMI, body mass index.

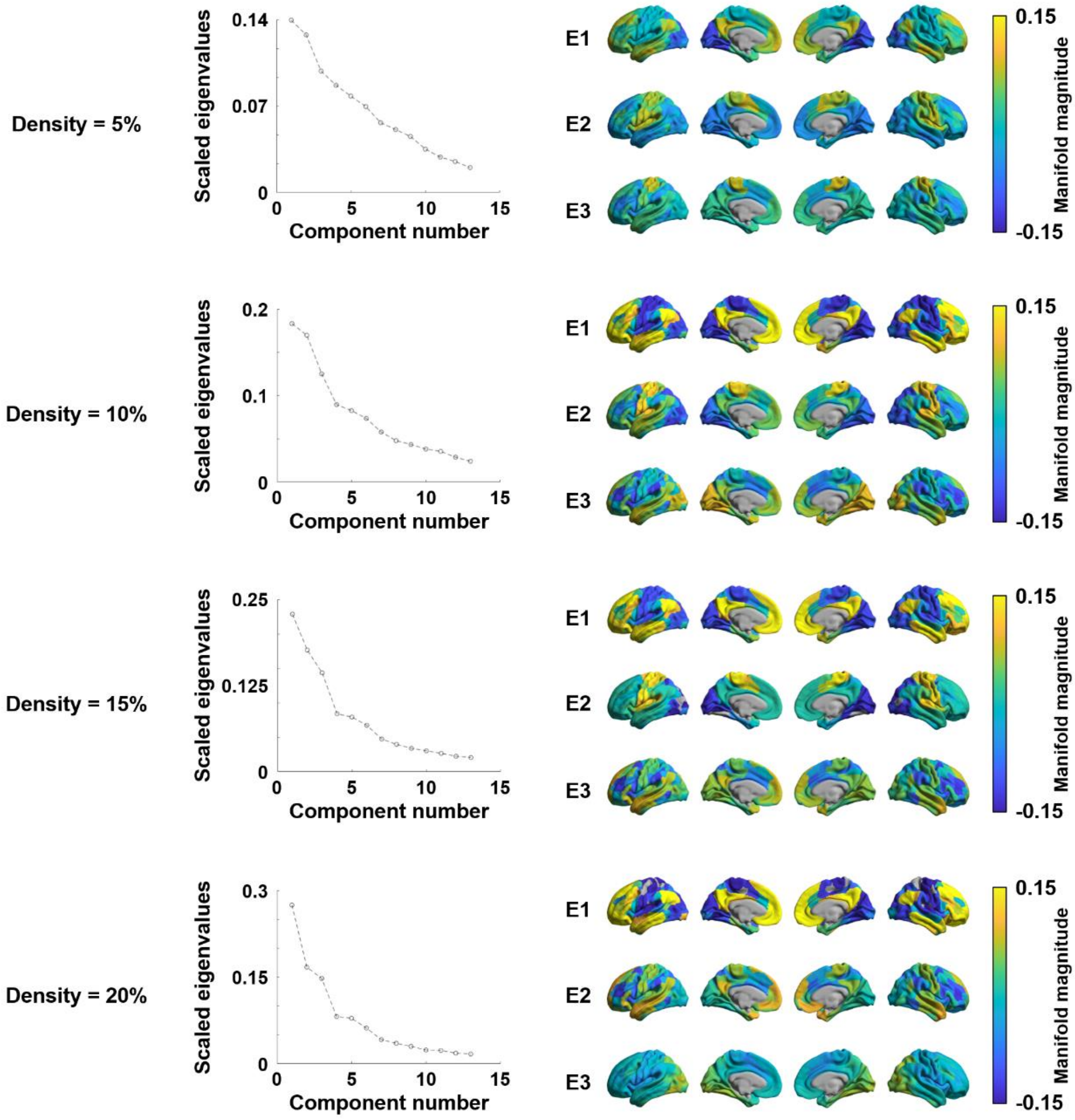

Supplementary Fig. 6: Functional manifolds with different connectome densities.

Connectome density from 5% to 20% with 5% interval was applied.

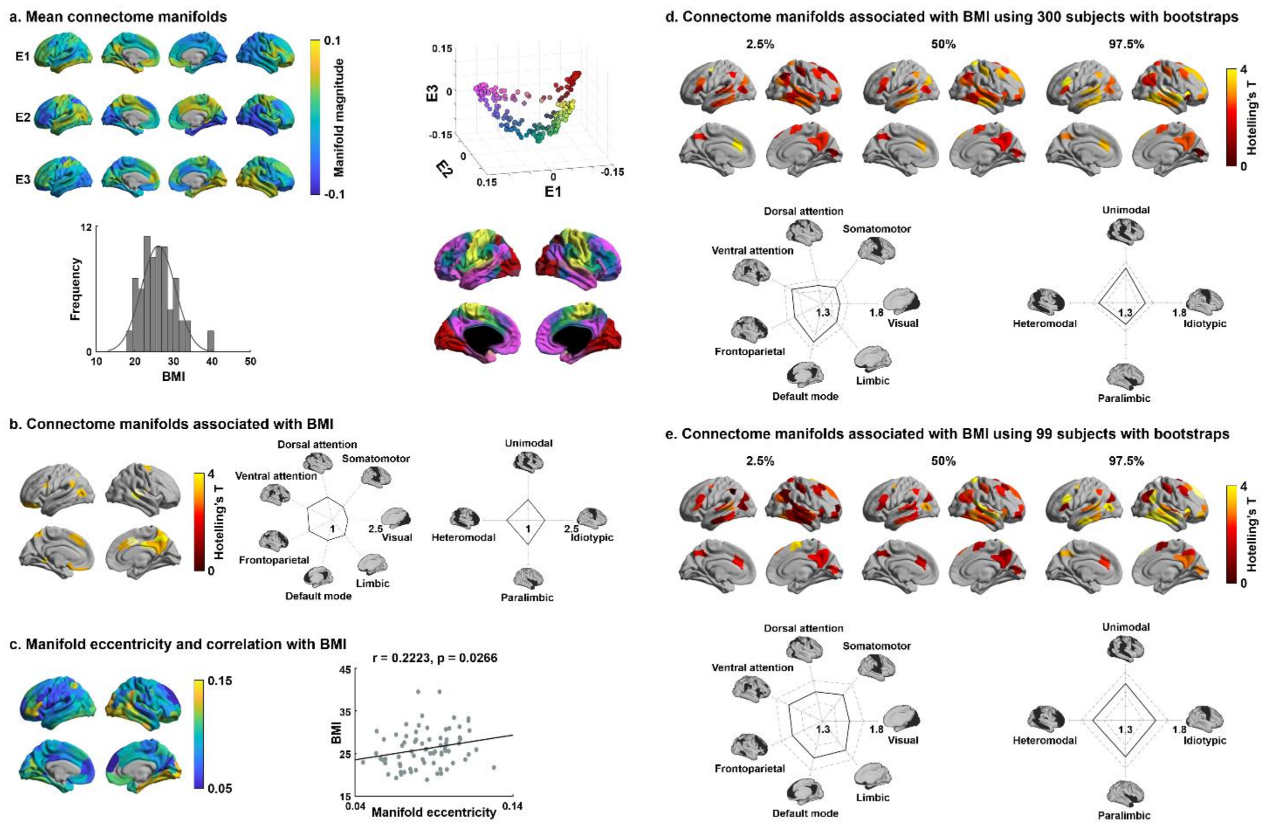

Supplementary Fig. 7: Reproducibility in HCP validation dataset.

(a)–(c) Replication results using an independent dataset from the HCP S1200. For details, see *Fig. 1* and *S1*. **(d)** Results based on 300 randomly selected participants from incorporated dataset of HCP S900 and S1200. The t-statistics that showed mean value of 2.5, 50, and 97.5% across bootstraps are reported on brain surfaces. In the spider plots, mean and SD of t-statistics across bootstraps are reported with black solid and gray dotted lines. **(e)** Results using data of the remaining 99 participants. *Abbreviations:* BMI, body mass index.

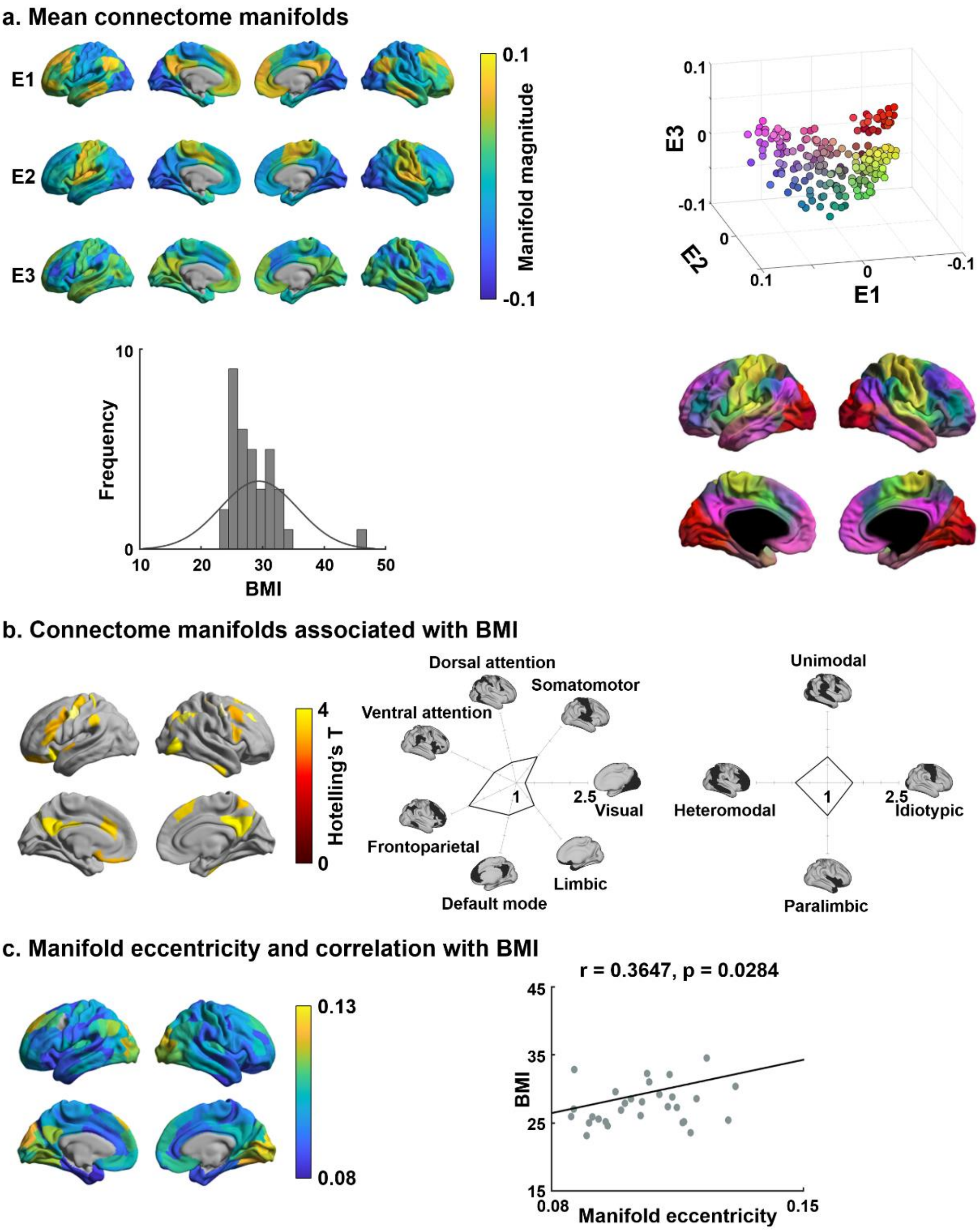

Supplementary Fig. 8: Replication results using an independent dataset obtained from St. Vincent's Hospital.

For details, see *Fig. 1* and *Supplementary Fig. 1*. *Abbreviations:* BMI, body mass index.

Supplementary references

1. Yeo, B. T. T. *et al.* The organization of the human cerebral cortex estimated by intrinsic functional connectivity. *J. Neurophysiol.* **106**, 1125–1165 (2011).

2. Blondel, V. D., Guillaume, J. L., Lambiotte, R. & Lefebvre, E. Fast unfolding of communities in large networks. *J. Stat. Mech. Theory Exp.* **2008**, (2008).

3. Mesulam, M. M. From sensation to cognition. *Brain* **121**, 1013–1052 (1998).

4. Schaefer, A. *et al.* Local-Global Parcellation of the Human Cerebral Cortex from Intrinsic Functional Connectivity MRI. *Cereb. Cortex* **28**, 3095–3114 (2018).
