# Supplementary material for: Inter-individual body mass variations relate to fractionated functional brain hierarchies
Source: Commun Biol. 2021 Jun 14;4:735. doi: 10.1038/s42003-021-02268-x (PMC8203627; doi:10.1038/s42003-021-02268-x)
Supplement: Supplementary file 2 — Description of Supplementary Files [file 42003_2021_2268_MOESM2_ESM.pdf]

Description of Additional Supplementary Files

File name: Supplementary data 1

Description: Significant gene lists correlated with connectome manifolds associated with BMI.

File name: Supplementary data 2

Description: Source data for all graphs and charts.
