# Supplementary material for: Inter-individual body mass variations relate to fractionated functional brain hierarchies
Source: Commun Biol. 2021 Jun 14;4:735. doi: 10.1038/s42003-021-02268-x (PMC8203627; doi:10.1038/s42003-021-02268-x)
Supplement: Supplementary file 5 — Reporting Summary [file 42003_2021_2268_MOESM5_ESM.pdf]

nature research

Corresponding author(s): Bo-yong Park
Boris C. Bernhardt

Last updated by author(s): Apr 23, 2021

Reporting Summary

Nature Research wishes to improve the reproducibility of the work that we publish. This form provides structure for consistency and transparency in reporting. For further information on Nature Research policies, see our Editorial Policies and the Editorial Policy Checklist.

Statistics

For all statistical analyses, confirm that the following items are present in the figure legend, table legend, main text, or Methods section.

n/a	Confirmed	
[ ]	[x]	The exact sample size (*n*) for each experimental group/condition, given as a discrete number and unit of measurement
[x]	[ ]	A statement on whether measurements were taken from distinct samples or whether the same sample was measured repeatedly
[ ]	[x]	The statistical test(s) used AND whether they are one- or two-sided<br>*Only common tests should be described solely by name; describe more complex techniques in the Methods section.*
[ ]	[x]	A description of all covariates tested
[ ]	[x]	A description of any assumptions or corrections, such as tests of normality and adjustment for multiple comparisons
[ ]	[x]	A full description of the statistical parameters including central tendency (e.g. means) or other basic estimates (e.g. regression coefficient) AND variation (e.g. standard deviation) or associated estimates of uncertainty (e.g. confidence intervals)
[ ]	[x]	For null hypothesis testing, the test statistic (e.g. *F*, *t*, *r*) with confidence intervals, effect sizes, degrees of freedom and *P* value noted<br>*Give P values as exact values whenever suitable.*
[x]	[ ]	For Bayesian analysis, information on the choice of priors and Markov chain Monte Carlo settings
[x]	[ ]	For hierarchical and complex designs, identification of the appropriate level for tests and full reporting of outcomes
[ ]	[x]	Estimates of effect sizes (e.g. Cohen's *d*, Pearson's *r*), indicating how they were calculated

Our web collection on statistics for biologists contains articles on many of the points above.

Software and code

Policy information about availability of computer code

Data collection	We obtained the minimally processed imaging and phenotypic data from the HCP (https://www.humanconnectome.org/).
Data analysis	Data preprocessing: https://github.com/Washington-University/HCPpipelines & https://gitlab.com/by9433/funp<br>Connectome manifold generation: https://github.com/MICA-MNI/BrainSpace<br>Manifold eccentricity calculation: https://github.com/MICA-MNI/micaopen/tree/master/manifold_features<br>Graph measure calculation: https://sites.google.com/site/bctnet<br>Transcriptomic association analysis: https://neurovault.org; http://genetics.wustl.edu/jdlab/csea-tool-2; https://github.com/rmarkello/abagen

For manuscripts utilizing custom algorithms or software that are central to the research but not yet described in published literature, software must be made available to editors and reviewers. We strongly encourage code deposition in a community repository (e.g. GitHub). See the Nature Research guidelines for submitting code & software for further information.

Data

Policy information about availability of data

All manuscripts must include a data availability statement. This statement should provide the following information, where applicable:

- Accession codes, unique identifiers, or web links for publicly available datasets
- A list of figures that have associated raw data
- A description of any restrictions on data availability

The imaging and phenotypic data were provided, in part, by the Human Connectome Project, WU-Minn Consortium (https://www.humanconnectome.org/) and they are available after approval. Data from St. Vincent's Hospital are not publicly available due to IRB restrictions. The subsets of data from these databases that were used in the present work are available from the authors upon request. Source data are provided with this paper.

Field-specific reporting

Please select the one below that is the best fit for your research. If you are not sure, read the appropriate sections before making your selection.

[x] Life sciences [ ] Behavioural & social sciences [ ] Ecological, evolutionary & environmental sciences

For a reference copy of the document with all sections, see nature.com/documents/nr-reporting-summary-flat.pdf

Life sciences study design

All studies must disclose on these points even when the disclosure is negative.

Sample size	325 unrelated young and healthy adults from the HCP S900 release<br>74 unrelated healthy adults from the HCP S1200 release<br>36 healthy adults acquired from the St. Vincent's Hospital
Data exclusions	We obtained the minimally processed imaging and phenotypic data from the S900 release of HCP. We excluded participants who are genetically related (i.e., twin pairs; n = 461), and who did not complete full imaging data with acceptable image quality (i.e., less than one T1- and T2-weighted and four sessions of rs-fMRI; n = 169), resulting in a total of 325 participants (mean ± SD age = 28.56 ± 3.74 years; 55% female). The mean BMI of the participants was 26.30 kg/m2 with SD of 5.16, range = 16.65 – 47.76 kg/m2), and the proportion of underweight (BMI < 18.5 kg/m2), healthy weight (18.5 ≤ BMI < 25 kg/m2), overweight (25 ≤ BMI < 30), and obesity (BMI ≥ 30) was 6:143:113:63. We furthermore selected additional data from the S1200 release of HCP to replicate the findings (see Sensitivity and reproducibility analyses section). The same exclusion criteria were applied (twin pairs n = 144; without full imaging n = 18). A total of 74 participants (mean ± SD age = 28.08 ± 3.90 years; 34% female; mean ± SD BMI = 26.17 ± 4.39 kg/m2, range 18.89 – 39.47 kg/m2) were enrolled, and the ratio of healthy weight, overweight, and obesity was 30:29:15.
Replication	To replicate findings, we collected an independent dataset from an independent site (St. Vincent's Hospital (SVH): n = 36; mean ± SD age = 38.78 ± 10.52 years; 47% female; mean ± SD BMI = 29.38 ± 6.29 kg/m2, range 23.15 – 57.13 kg/m2).
Randomization	We performed multivariate association analysis between BMI and the first three eigenvectors, which explained approximately 50% in connectome information, with the model controlling for age and sex. We utilized SurfStat (http://www.math.mcgill.ca/keith/surfstat/) to fit linear models of the formula b0 + b1·Age +b2·Sex +b3·BMI to multivariate data Y (of the form number of subjects × number of brain regions × number of eigenvectors). Inference was based on Hotelling's t-test in each parcel, and multiple comparisons were corrected for using the FDR procedure.<br>We linearly correlated BMI and manifold eccentricity in regions identified by the multivariate analysis, controlling for age and sex. Significance was assessed using 5,000 permutation tests. A null distribution was constructed and the real correlation strength was deemed significant if it did not belong to the 95% of the distribution (two-tailed p < 0.05).<br>We calculated linear correlations between the manifold eccentricity and within-module degree and participation coefficient. The significance of the associations between manifold eccentricity and modular measures were assessed using 5,000 permutation tests by randomly shuffling subjects. Multiple comparisons across different modular parameters were corrected using FDR.<br>To validate whether the gene symbols passing FDR < 0.05 were derived by chance or not, we repeated the correlation analysis using 100 randomly rotated cortical maps of the multivariate association analysis. We, thus, constructed a null distribution of spatial correlations between the expression patterns of the identified gene list and randomly rotated maps. The actual correlation t-statistic was placed into this null distribution to assess significance, and findings were again FDR-corrected.
Blinding	N/A

Reporting for specific materials, systems and methods

We require information from authors about some types of materials, experimental systems and methods used in many studies. Here, indicate whether each material, system or method listed is relevant to your study. If you are not sure if a list item applies to your research, read the appropriate section before selecting a response.

Materials & experimental systems

n/a	Involved in the study	
[x]	[ ]	Antibodies
[x]	[ ]	Eukaryotic cell lines
[x]	[ ]	Palaeontology and archaeology
[x]	[ ]	Animals and other organisms
[ ]	[x]	Human research participants
[x]	[ ]	Clinical data
[x]	[ ]	Dual use research of concern

Methods

n/a	Involved in the study	
[x]	[ ]	ChIP-seq
[x]	[ ]	Flow cytometry
[ ]	[x]	MRI-based neuroimaging

Human research participants

Policy information about studies involving human research participants

Population characteristics	325 unrelated young and healthy adults from the HCP S900 release: mean ± SD age = 28.56 ± 3.74 years; 55% female; mean ± SD BMI = 26.30 ± 5.16 kg/m2 (range = 16.65 – 47.76 kg/m2); proportion of underweight (BMI < 18.5 kg/m2), healthy weight (18.5 ≤ BMI < 25 kg/m2), overweight (25 ≤ BMI < 30), and obesity (BMI ≥ 30) = 6:143:113:63.<br>Additionally 74 participants from the S1200 release of HCP: mean ± SD age = 28.08 ± 3.90 years; 34% female; mean ± SD BMI = 26.17 ± 4.39 kg/m2 (range 18.89 – 39.47 kg/m2); the ratio of healthy weight, overweight, and obesity = 30:29:15.<br>36 independent dataset from St. Vincent's Hospital: mean ± SD age = 38.78 ± 10.52 years; 47% female; mean ± SD BMI = 29.38 ± 6.29 kg/m2 (range 23.15 – 57.13 kg/m2)
Recruitment	We obtained the minimally processed imaging and phenotypic data from the HCP. Additional data were collected from St. Vincent's Hospital.
Ethics oversight	Participant recruitment procedures and informed consent forms, including consent to share de-identified data, were previously approved by the Washington University Institutional Review Board as part of the HCP.<br>Data collection and usage were approved from the Institutional Review Boards of the Catholic University of Korea (no. XC15DIMI0012, approved March 2015), and written and informed consent was obtained from all participants.

Note that full information on the approval of the study protocol must also be provided in the manuscript.

Magnetic resonance imaging

Experimental design

Design type	Resting-state
Design specifications	Resting-state
Behavioral performance measures	Resting-state

Acquisition

Imaging type(s)	T1-weighted, resting-state fMRI
Field strength	3T
Sequence & imaging parameters	a) HCP: The T1-weighted images were acquired using a magnetization-prepared rapid gradient echo (MPRAGE) sequence (repetition time (TR) = 2,400 ms; echo time (TE) = 2.14 ms; field of view (FOV) = 224 × 224 mm2; voxel size = 0.7 mm3; and number of slices = 256). The T2-SPACE sequence was used for scanning T2-weighted structural data, and the acquisition parameters were the same as the T1-weighted data except for the TR (3,200 ms) and TE (565 ms). The rs-fMRI data were collected using a gradient-echo EPI sequence (TR = 720 ms; TE = 33.1 ms; FOV = 208 × 180 mm2; voxel size = 2 mm3; number of slices = 72; and number of volumes = 1,200).<br>b) SVH: The T1-weighted images were acquired using a MPRAGE sequence (TR = 1,900 ms; TE = 2.49 ms; FOV = 250 × 250 mm2; voxel size = 1 mm3; and number of slices = 160). The rs-fMRI data were collected using a gradient-echo EPI sequence (TR = 2,490 ms; TE = 30 ms; FOV = 220 × 220 mm2; voxel size = 3.4 × 3.4 × 3 mm3; number of slices = 36; and number of volumes = 150).
Area of acquisition	whole brain

Diffusion MRI ☐ Used ☒ Not used

Preprocessing

Preprocessing software	T1: FSL, FreeSurfer, and Workbench (gradient nonlinearity and b0 distortion correction; co-registration between T1 and T2; bias field correction; registration to MNI; surface generation; spherical registration to Conte69)<br>Rs-fMRI: FSL, FreeSurfer, and Workbench (EPI distortions and head motion correction; registered to the T1-weighted data and subsequently to MNI152 space; magnetic field bias correction; skull removal; intensity normalization; noise components removal; map to standard grayordinate space, with a cortical ribbon-constrained volume-to-surface mapping algorithm)
Normalization	Rs-fMRI ddata were co-registered to T1-weighted data in MNI152 space. The rs-fMRI data were mapped to subject-specific midthickness surfaces and resampled to the Conte69 template.
Normalization template	MNI template in volume space and Conte69 template in surface space
Noise and artifact removal	Noise components attributed to head movement, white matter, cardiac pulsation, arterial, and large vein related contributions were removed using FMRIB's ICA-based X-noiseifier (ICA-FIX)
Volume censoring	volumes with frame-wise displacement > 0.5 mm were removed

Statistical modeling & inference

Model type and settings	Multivariate analyses controlled for age and sex
Effect(s) tested	Hotelling's T and FDR corrected p-values

Specify type of analysis: ☐ Whole brain ☐ ROI-based ☒ Both

Anatomical location(s)	Schaefer parcellation
Statistic type for inference (See Eklund et al. 2016)	ROI-wise
Correction	FDR

Models & analysis

n/a	Involved in the study
☐	☒ Functional and/or effective connectivity
☐	☒ Graph analysis
☐	☒ Multivariate modeling or predictive analysis

Functional and/or effective connectivity	Pearson's correlation
Graph analysis	Individual-level weighted graph<br>We calculated within-module degree and participation coefficient. Modules were defined using established intrinsic functional communities, a Louvain community detection algorithm, and a schema of cortical hierarchy.
Multivariate modeling and predictive analysis	We performed multivariate association analysis between BMI and the first three eigenvectors, which explained approximately 50% in connectome variance, with the model controlling for age and sex. We corrected for multiple comparisons using the FDR procedure.
